# Retinoblastoma in times of war: changing patterns of presentation, treatment, and prognosis in Gaza

**DOI:** 10.3389/fonc.2025.1747870

**Published:** 2026-01-14

**Authors:** Yacoub A. Yousef, Mohammad Msallam, Mona Mohammad, Hadeel Halalsheh, Jakub Khzouz, Maysa Al-Hussaini, Imad Jaradat, Munir Shawagfeh, Iyad Sultan, Mustafa Mehyar, Ibrahim Al-Nawaiseh, Asem Mansour

**Affiliations:** 1 Departments of Surgery, King Hussein Cancer Centre (KHCC), Amman, Jordan; 2 Gaza Eye Hospital, Gaza, Palestine; 3 Departments of Pediatrics Oncology, King Hussein Cancer Centre (KHCC), Amman, Jordan; 4 Departments of Pathology and Laboratory Medicine, King Hussein Cancer Centre (KHCC), Amman, Jordan; 5 Departments of Cell Therapy and Applied Genomics, King Hussein Cancer Centre (KHCC), Amman, Jordan; 6 Departments of Radiation Oncology, King Hussein Cancer Centre (KHCC), Amman, Jordan; 7 Departments of Anesthesia, King Hussein Cancer Centre (KHCC), Amman, Jordan; 8 Departments of Diagnostic Radiology, King Hussein Cancer Centre (KHCC), Amman, Jordan

**Keywords:** delayed diagnosis, Gaza war, humanitarian crisis, pediatric cancer, retinoblastoma

## Abstract

**Background:**

Retinoblastoma (Rb) is a curable childhood eye cancer when treated early, yet survival remains far lower in developing countries than developed countries. Delayed access to specialized care is a key barrier, and armed conflicts further worsen outcomes. Since October 2023, Gaza has endured a devastating war and complete siege, leading to health system collapse, medicine shortages, and restricted movement. This study examined the impact of war-related delays on presentation, treatment, and outcomes in Gaza’s Rb patients.

**Methods:**

We retrospectively analyzed 12 children (17 eyes) with retinoblastoma treated at King Hussein Cancer Center (2016–2025), grouped into pre-war (2016–Oct 2023; 7 patients, 10 eyes) and during-war (Oct 2023– Oct 2025; 5 patients, 7 eyes) cohorts. Data included presenting signs, lag time, stage, treatment, eye salvage, metastasis, and survival.

**Results:**

Median lag-time increased from 30 days before the war to 180 days (range 120-270) during the siege. Disease severity was significantly worse: before the war, only 20% of eyes were group E and none had extraocular disease, whereas during the war 57% were group E and 29% presented with extraocular extension(p=0.036). Eye salvage dropped dramatically, from 70% pre-war to 29% during the war(p=0.046). Primary enucleation rose nearly threefold, from 20% before the war to 57% during the siege, and 20% in the war had bilateral enucleation. Critically, while no patient in the pre-war cohort developed metastasis or died, 40% of children during the war developed metastasis and 20% died.

**Conclusion:**

The siege during the Gaza war had a devastating impact on outcomes. It substantially increased the risk of death from retinoblastoma; an otherwise highly curable childhood cancer when timely treatment is available. Outcomes were dictated not by medical limitations but by political and humanitarian barriers. Urgent international action is essential to secure humanitarian corridors, safeguard children’s right to timely cancer treatment, and ensure healthcare is protected from the effects of war.

## Introduction

Globally, the incidence of retinoblastoma (Rb) is approximately one in 15,000 to 20,000 live births (1, 2). Disease-specific mortality has markedly improved over the past years (3), for example in Jordan the mortality rate decreased from 38% to 5% (4), however, global disparities in regional mortality rates remain still an issue (5, 6). The global Retinoblastoma Study group showed that children with Rb in a low-income country are at 16 times higher risk of dying at any time within three years of diagnosis than those in high-income countries (7), due to delayed diagnosis and delayed access to adequate health care, therefore, timely diagnosis and prompt management for Rb, the most common primary intraocular malignancy in children, are critical for cure (8, 9).

A numerous international initiatives are dedicated to reducing mortality for Rb in the developing countries; the Eye Cancer Foundation supports fellowships for ophthalmologists from underserved regions, enabling them to establish local eye cancer programs, while the International Retinoblastoma Comprehensive Course (IRBCC) delivers free online training to strengthen global diagnostic and treatment capacity. St. Jude Children’s Research Hospital promotes early detection and telemedicine through Cure4Kids and ORBIS Web, with the aim of achieving 70% survival worldwide by 2030, and there are much more. In addition, large collaborative research networks, including the American Joint Committee on Cancer Ophthalmic Oncology Task Force, the Global Retinoblastoma Study Group, and the High-Risk Retinoblastoma Collaborative Study Group work collectively to improve understanding of retinoblastoma, establish consensus guidelines, and standardize management worldwide (4–7, 10–12). On the other hand, some political actions, such as armed conflict and siege, undermine these advances.

Previously, Gazan retinoblastoma patients treated at King Hussein Cancer Center showed no differences in tumor stage or outcomes compared with other patients from the region (4). Notably, none of the known Gazan patients treated during the five-year period before October 2023 developed metastasis or died from the disease. However, a catastrophic humanitarian crisis has occurred in Gaza because of the war and siege since October 2023. Reports indicate that by September 2025, more than 64,000 civilians have been killed, including over 18,500 children (13, 14), that is much higher than the global annual incidence of Rb (6,275 new cases worldwide) (15).

Normally, children from Gaza require referral to nearby countries like Jordan, for diagnosis and treatment. Previous research has shown that diagnostic delays in Rb lead to more advanced disease at presentation, lower eye salvage, and worse survival outcomes, particularly in low- and middle-income countries (7, 12, 16), Under the current siege, with restrictions on movement, limited access to medications, and collapse of healthcare infrastructure, these delays are expected to be longer and their consequences on eye salvage and survival are more severe.

This study aims to evaluate the impact of the war and the siege in Gaza on the lag time between the first sign of Rb and access to specialized care. Herein, we evaluate the patterns of presentation and management outcomes among patients from Gaza diagnosed with Rb who underwent treatment at King Hussein Cancer Center (KHCC), in Amman, Jordan, during the war. These patterns were compared with previously treated Rb Gazan children at the same cancer center (KHCC) before the war.

## Materials and methods

The Institutional Review Board at KHCC (25KHCC262) approved the current study. It was a retrospective, clinical case series of 12 children from Gaza with 17 eyes affected with Rb, who had been managed at KHCC before and during the war in Gaza that started on October 7, 2023. Data from children managed between 2016 and 2025 was analyzed.

All patients included in this study had complete information for the primary outcome measures, including lag time, disease stage at presentation, eye salvage, metastatic status, and survival. Patients with incomplete data for these key outcomes were not included in the analysis. Missing ancillary variables, when present, were reported descriptively and did not affect the primary outcome analyses.

### Patients grouping

Gazan retinoblastoma cases from 2016–October 2023 were grouped as the “pre-war” cohort, a period during which access to care was stable and consistent. Children were routinely referred outside Gaza for diagnosis and treatment through an established medical evacuation system, which did not undergo major structural changes over these years. No significant shifts in referral policies, treatment availability, or access to specialized oncology services occurred prior to October 2023 that would meaningfully affect patterns of presentation or outcomes. The disruption in care began after October 2023, when the war halted medical evacuations, restricted access to specialized care, and caused prolonged delays in diagnosis and treatment. Therefore, cases before October 2023 represent a relatively stable baseline and serve as an appropriate comparator for the “war-period” cohort.

### Inclusion criteria

Inclusion criteria included children who are citizens of Gaza who had a clinical and/or pathological diagnosis of Rb and were managed at KHCC and followed for at least 1 year after diagnosis, or who are still under active treatment and/or follow-up. Patients who were not citizens of Gaza were excluded from this study.

### Clinical data and treatment modalities

Data collected included age at diagnosis, sex, laterality, affected site, family history, International Intraocular Retinoblastoma Classification stage (IIRC) at diagnosis (17), presenting signs and symptoms, the lag time between the presenting sign and starting treatment, modality of treatment, eye salvage, metastasis, and mortality. Selection and data collection required access to medical records and Ret-Cam images. Lag time was defined as the interval between the time parents first noticed a clinical sign suggestive of retinoblastoma (most commonly leukocoria) and the initiation of treatment. This information was obtained from parental reports as documented in the medical records at the time of presentation.

Diagnosis and staging were based on clinical examination under general anesthesia and supported by ocular ultrasonography and MRI at the time of diagnosis. For patients with intraocular Rb, we used a combination chemotherapy regimen of CVE (carboplatin, vincristine, and etoposide). Each CVE cycle was repeated every 4 weeks for a total of 6-8 cycles according to the patient’s condition and tumor status.

Ocular oncology follow-up was provided with examination under anesthesia before every cycle of chemotherapy and every 4 weeks thereafter. Fundus images were obtained using a RetCam system (RetCam II, Clarity Medical Systems, and RetCam, Natus Medical Incorporated; Pleasanton, CA, USA).” Combination focal therapy was applied as needed as Trans pupillary thermotherapy (TTT) and/or triple freeze-thaw cryotherapy (MIRA CR 4000).

For patients presenting with clinically evident extraocular extension, neoadjuvant chemotherapy was initiated prior to enucleation and subsequent radiotherapy. In cases with radiological evidence of resectable optic nerve invasion, primary enucleation was performed as the initial treatment, followed by adjuvant systemic chemotherapy if they have high-risk pathological features—including massive choroidal invasion, post-laminar optic nerve invasion, anterior segment invasion, and scleral involvement—were identified. Those with extrascleral extension or a positive optic nerve margin received external beam radiotherapy (EBRT) after doing bone marrow and LP to rule out metastasis.

All Group E eyes were immediately enucleated, except in cases where the fellow eye was also deemed unsalvageable (Group E) or exhibited extraocular extension; in such situations, conservative therapy was considered as an alternative to enucleation. External beam radiation therapy was administered when needed consistently by applying 45 Gy in 25 fractions.

### Statistical analysis

Patient-level analyses were used for demographic characteristics, lag time, metastatic status, and survival outcomes, whereas eye-level analyses were used for tumor classification, treatment modality, and eye salvage outcomes. Descriptive analysis was carried out using the mean, median, and range. Comparative analysis was carried out between patients treated before and during the war. The p-value was measured using the Fisher’s exact tests to analyze each factor’s predictive power.

Due to the small sample size and the limited number of outcome events, more advanced statistical approaches such as multivariable regression or time-series analyses were not feasible. Therefore, descriptive statistics and simple comparative analyses were considered the most appropriate and methodologically sound approach for this exploratory study.

## Results

Over a 10-year period (2016-2025), 12 children from Gaza who had 17 eyes with Rb were treated at KHCC and met our inclusion criteria. The mean and median age at diagnosis were 26 months; seven (58%) were male, five (42%) had bilateral disease, and only two (17%) had positive family history for Rb. The overall median lag time between signs of disease and starting treatment was 40 days. The most common presenting sign was leukocoria in eight (67%) patients; however, two (17%) presented with buphthalmos, and one (8%) presented with gross orbital disease (**Table 1**). The median follow-up was 36 months for the pre-war group and 9 months for the during-war group. RB1 germline testing performed on peripheral blood samples showed that all patients with bilateral retinoblastoma had germline RB1 mutations, whereas no germline mutations were detected in patients with unilateral disease. Only one (with bilateral Rb) had positive family history of Rb.

**Table 1:** Demographics and presenting symptoms 12 Rb patients from Gaza before and during the Gaza war.

Feature		Total	%	Before	%	After	%	P value
Number of patients		12 Patients		7	58	5	42	
Number of eyes		17 Eyes		10	59	7	41	
Age at diagnosis median, mean, range (Months)		26, 25, 3-48 months		25, 24, 3-44 months		28, 27, 12-48 months		
Gender	Male	7	58	4	57	3	60	0.92
	Female	5	42	3	43	2	40	
Laterality	Bilateral	5	42	3	43	2	40	0.461
	Unilateral	7	58	4	57	3	60	
Family history	Positive	2	17	1	14	1	20	0.396
	Negative	10	83	6	86	4	80	
Side	Right	9	53	5	50	4	57	0.386
	Left	8	47	5	50	3	43	
Presenting symptom	Leukocoria	8	67	6	86	2	40	0.009
	Squint	1	8	1	14	0	0	
	Buphthalmous	2	17	0	0	2*	40	
	Extraocular	1	8	0	0	1*	20	
Delay for treatment median, mean, range (Days)		40, 91, 15-270 days		30,31,15-60 days		180,175, 120-270 days		

*Parents for all these three patients initially noticed leukocoria, but because of the delay to access medical care, they 2 had buphthalmous and 1 had orbital disease at time of treatment.

### Rb children before the Gaza war

Out of seven patients, 4 (57%) were males, 3 (43%) had bilateral disease, 1 (14%) had a family history of Rb, and the median age at diagnosis was 24 months. The median lag time between signs of disease and starting treatment was 30 days. The most common presenting sign was leukocoria in six (86%) patients, and no one presented with buphthalmous or extraocular disease (**Table 1**).

**Figure 1:**
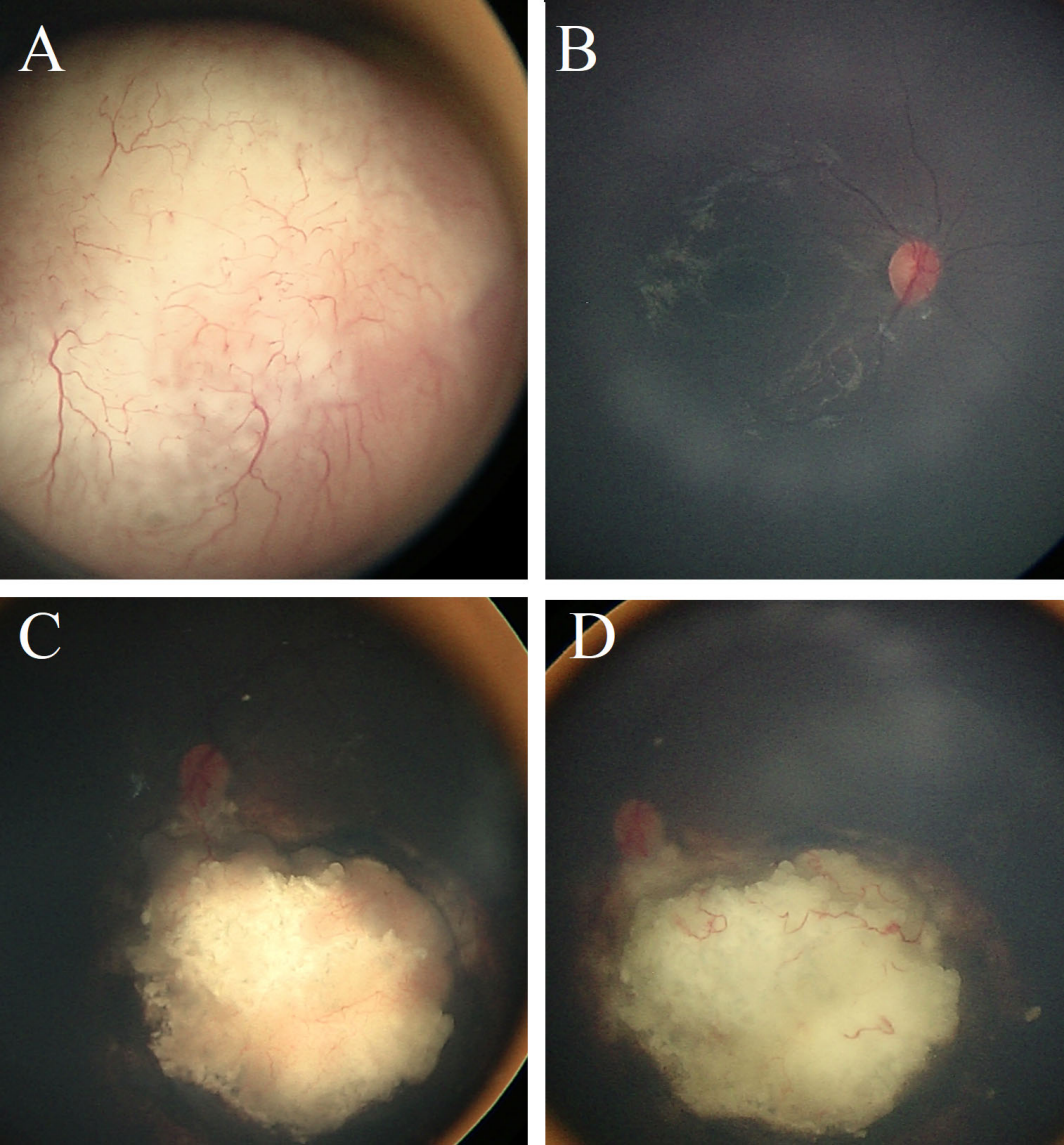
Retinoblastoma before the Gaza war. Clinical course of a 2-year-old Gazan girl before the war who presented with left leukocoria and was diagnosed with unilateral retinoblastoma (Group D) **(A)**, while the right eye was normal **(B)**. Complete tumor regression was achieved after six cycles of CVE chemotherapy with focal consolidation using transpupillary thermotherapy (TTT). However, significant recurrence was observed three months later **(C)**, which was treated with I-125 radioactive plaque therapy. No additional treatment was given thereafter, and at three years post-plaque therapy the tumor remained inactive with no signs of recurrence **(D)**.

Out of 10 affected eyes, eight (80%) eyes were collectively IIRC group B, C, or D, 2 (20%) eyes were group E, and no one had extraocular disease (**Table 2**). The two (20%) eyes that were in group E were managed by primary enucleation, while the other eight (80%) received conservative therapy as a combination of systemic chemotherapy with focal consolidation therapy. At the last date of follow-up, seven (70%) eyes were successfully salvaged, three (30%) were enucleated, and no one had metastasis or was dead (**Figure 1**, **Table 2**).

**Table 2:** Tumor features, treatment and management outcomes for 12 Rb patients from Gaza before and during the Gaza war.

Feature		Total	%	Before	%	After	%	P value
Number of patients		12 Patients		7	58	5	42	
Number of eyes		17 Eyes		10	59	7	41	
Stage	Intraocular	15	88	10	100	5	71	0.036
	Extraocular	2	12	0	0	2	29	
IIRC	A	0	0	0	0	0	0	0.029
	B	1	6	1	10	0	0	
	C	2	12	2	20	0	0	
	D	6	35	5	50	1	14	
	E	6	35	2	20	4	57	
	Extraocular	2*	12	0	0	2*	29	
Primary treatment	Enucleation	6	35	2	20	4	57	0.114
	Conservative	11	65	8	80	3^&^	43	
Eye salvage **	Yes	9	53	7	70	2	29	0.046
	No	8	47	3	30	5	71	
Bilateral Enucleation**	Yes	1	8	0	0	1	20	0.108
	No	11	92	7	100	4	80	
Metastasis	Yes	2	17	0	0	2	40	0.034
	No	10	83	7	100	3	60	
Survival	Alive	11	92	7	100	4	80	0.108
	Dead	1	8	0	0	1^@^	20	

**Figure 2:**
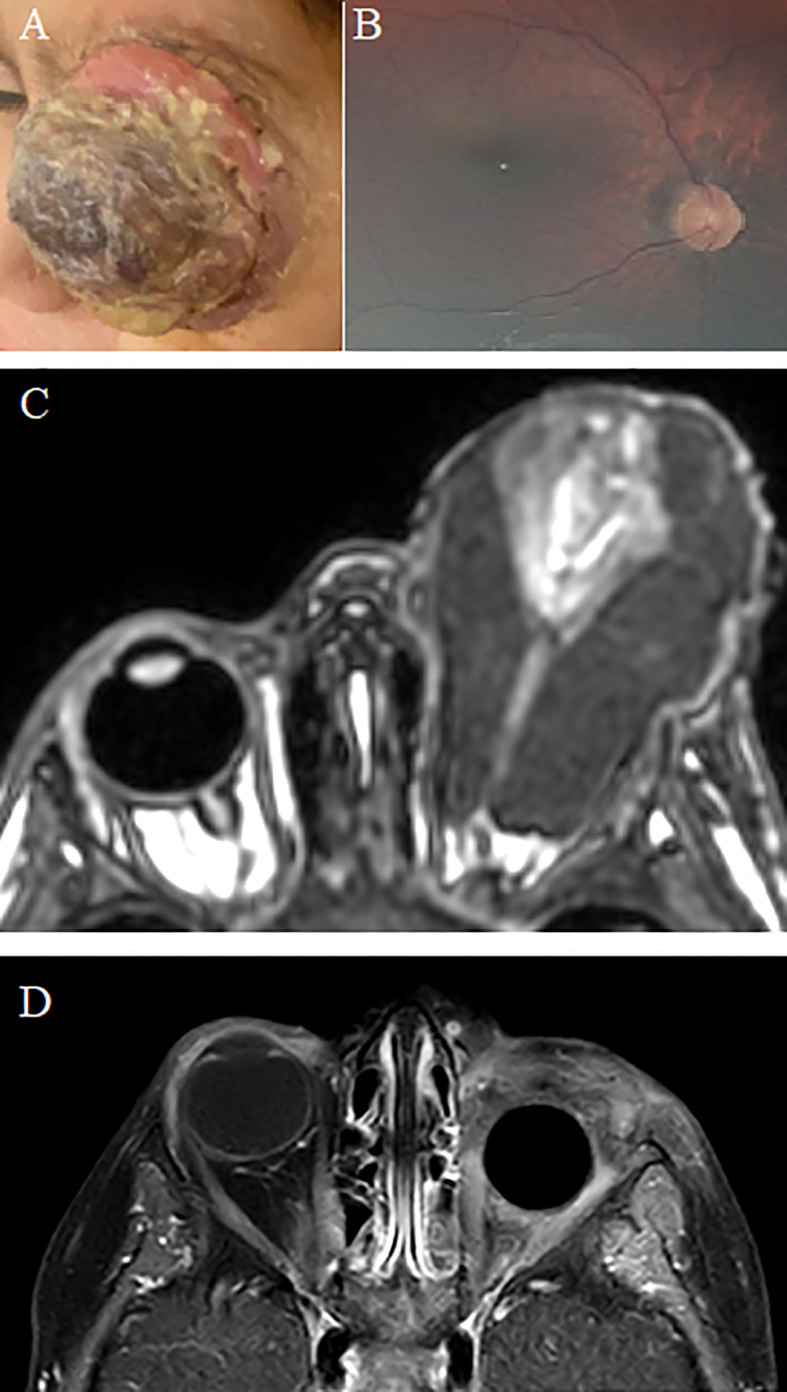
Retinoblastoma during the Gaza war (Unilateral with Orbital Extension). A child with left unilateral retinoblastoma presented 180 days after the first sign (leukocoria). At presentation, there was a large left orbital mass **(A)** with a normal right fundus **(B)**. MRI confirmed an extensive left orbital retinoblastoma **(C)**, and he had concomitant bone marrow metastasis. The patient underwent systemic chemotherapy, left eye enucleation, orbital radiotherapy, and bone marrow transplantation. Post-treatment MRI shows enucleation with orbital implant in place **(D)**.

*Two patients had extraocular tumor at presentation. One had gross orbital invasion (**Figure 2**), and one had post laminar optic nerve invasion detected in MRI and confirmed by pathology.

^&^ Out of these 3 eyes; 1 was group D and 2 were group E. These 2 group E eyes were offered conservative treatment because the other eye was enucleated immediately at presentation (one had optic nerve invasion in MRI, and the other one had very advanced tumor with phthisis and massive intraocular hemorrhage in a non-salvageable eye).

**^@^**This patient passed away with CNS metastasis. However, another patient had bone marrow metastasis and treated by bone marrow transplantation. Three months after BMT this child still alive and free of disease, however, it is too early to decide regarding survival.

**The total number of patients was 12, only one had bilateral enulceation (2 eyes), 6 patients had one eye enucleated, and 5 patients had no eye with enucleated.

Note: Patient-level analyses were used for demographic characteristics, lag time, metastatic status, and survival outcomes, whereas eye-level analyses were used for tumor classification, treatment modality, and eye salvage outcomes.

### Rb children during the Gaza war

Out of five patients, three (60%) were males, two (40%) had bilateral disease, one (20%) had a family history of Rb, and the median age diagnosis was 28 months. The median lag time between signs of disease and starting treatment was 180days (range 120-270 days). Leukocoria was the presenting sign in two (40%) patients, while two (40%) patients presented with buphthalmos, and one (20%) presented with orbital disease (**Table 1**).

**Figure 3:**
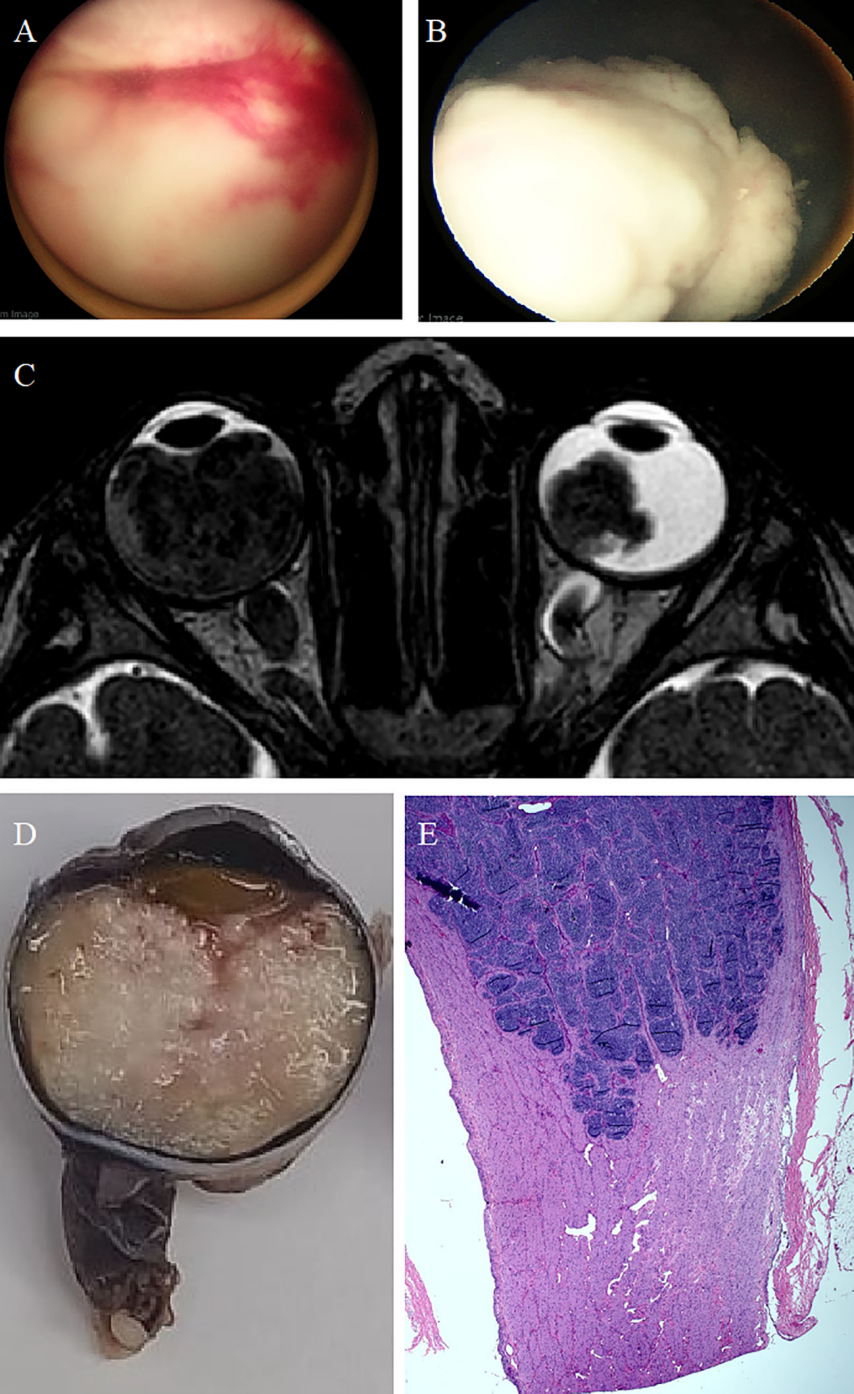
Bilateral Retinoblastoma during the Gaza War with CNS Metastasis. A 2-year-old girl presented with bilateral retinoblastoma. The right eye **(A)** had a massive intraocular tumor filling the globe, while the left eye **(B)** showed advanced group E disease with vitreous seeding and neovascularization of the iris. MRI **(C)** confirmed post-laminar optic nerve invasion in the right eye (extraocular disease), necessitating primary enucleation. Histopathology revealed tumor filling the entire eye globe **(D)** and infiltration of the optic nerve extending 11 mm posteriorly, reaching up to 7 mm from the transected resection margin **(E)**. Despite treatment, the patient later developed CNS metastasis and died.

Out of seven affected eyes, one (14%) eye was IIRC group D, four (57%) eyes were group E, and two (29%) eyes had extraocular disease (one with a clinical orbital mass (**Figure 2**), and the second one had massive optic nerve (**Figure 3**) invasion detected in MRI) (**Table 2**). Conservative therapy with the aim of saving the eye globe was offered for three (43%) eyes, including two eyes in group E (because the other eye was not salvaged).

Three (43%) eyes underwent primary enucleation, including two group E eyes and one eye with MRI evidence of massive optic nerve invasion. Moreover, the child with orbital disease (**Figure 2**) was treated by neoadjuvant chemotherapy, followed by enucleation and radiation therapy, but since he developed lymphatic and bone marrow metastasis, bone marrow transplant (BMT) was performed.

At the last date of follow-up, two (29%) eyes were successfully salvaged, five (71%) eyes were enucleated (including one patient with bilateral enucleation) and 2 (40%) patients had distant metastasis, and one of them (20%) had CNS metastasis and succumbed to her disease (**Table 2**).

### Comparison between Rb children before and during the Gaza war

Overall, the eye salvage rate decreased from 7 of 10 eyes pre-war (70.0%; 95% CI, 34.8–93.3) to 2 of 7 eyes during the war period (28.6%; 95% CI, 3.7–71.0). No patients in the pre-war cohort developed metastatic disease (0/7; 0.0%; 95% CI, 0.0–41.0), whereas metastasis occurred in 2 of 5 patients during the war (40.0%; 95% CI, 5.3–85.3). All pre-war patients survived (7/7; 100%), compared with 4 of 5 patients during the war period (80.0%), corresponding to a mortality rate of 20.0% (95% CI, 0.5–71.6).

There was no difference between patients before the war and during the war in terms of sex, laterality, and incidence of familial diseases. The median age at diagnosis for patients during the war was 3 months older than those before the war. Patients were more likely to present with signs of advanced ocular disease (buphthalmous) rather than leukocoria that was the most common presentation for patients before the war. The median lag time between the signs of Rb and accessibility to treatment was 180 days during the war which is 5 months longer compared to the lag time before the war (**Table 1**).

During the war, patients presented with a more advanced stage; 29% presented with extra ocular disease compared to none, (p=0.036) during and before the war, respectively Overall, 86% of eyes during the war were classified as group E or had extraocular disease at diagnosis compared to 20% of eyes in group E before the war (p= 0.046) (**Table 2**).

The overall eye salvage rate during the war was 29% compared to 70% before the war (p=0.046). Even though the difference in the usage of enucleation as primary treatment was not statistically significant (57% vs 20%, p=0.114), patients during the war mandated the team to attempt conservative therapy for group E eyes (that are usually treated by primary enucleation) in a trial to save some vision because these patients lost the other eye due to very advanced disease. Twenty percent of patients during the war ended up with bilateral enucleation while none before the war had bilateral enucleation. At the last date of follow-up, 40% of the patients during the war had metastasis, and 20% were dead, compared to not a single case of metastasis in patients treated before the war (**Table 2**).

## Discussion

During prolonged conflicts accompanied by a complete blockade, such as is the situation in Gaza, priorities for survival shift. Resources and international aid are directed primarily toward basic necessities like food and shelter, rather than priority for care for rare diseases like Rb. This results in serious implications for children with Rb, which is a life-threatening but highly curable condition when treated promptly (2, 4, 5, 17). While successful treatment of Rb depends on early diagnosis and immediate access to specialized therapy, under the current tight siege, movement outside Gaza is highly restricted, and many children suffer from long delays before referral, if referral is even a possibility. Consequently, children present at advanced stages, when the likelihood of eye and life salvage is much lower. As a consequence, many Gazan children lose their vision, and some die, not because effective treatment is unavailable, but because access is denied. Physicians in Gaza and referral centers face moral, ethical and emotional challenges; acknowledging that while Rb is a potentially curable cancer, yet the current war, the demolished health systems, and political restrictions dictate outcomes more than medicine itself.

Rb patients with delayed diagnosis and referral are expected to present with a more advanced tumor stage (17–20). In this study, the average lag time between signs of disease and access to treatment for Rb patients from Gaza during the war was 180 days (6 months) compared to 30 days for the same community before the war, and this was reflected clearly and significantly in the presenting signs at time of treatment. During the siege, 57% (n=4/7) of the affected eyes had very advanced intraocular stage (staged as group E), and 29% (n=2/7) had an extraocular disease at the time of treatment, while only 20% (n=2/10) of the affected eyes before the war were in group E, and none had an extraocular disease. During the siege, patients were more likely to present with extraocular disease or with advanced intra ocular disease with a significant p value of 0.036 and 0.029, respectively, which resulted in reducing the chance for eye globe salvage (70% before the war vs. 29% during the war; p= 0.046). Unfortunately, patient survival outcomes deteriorated significantly during the war as well. Tragically, 40% of patients diagnosed during the conflict presented with metastatic disease, and 20% have died, whereas no patients in the pre-war cohort developed metastasis or died.

The signs and symptoms of Rb depend on its size and location. Leukocoria is the most frequent presenting sign of Rb, reported in approximately 50–60% of cases, followed by strabismus (25%) and inflammatory signs (6–10%) (20–23). Previously, we found that 53% of Syrian refugees’ with Rb presented with leukocoria, followed by strabismus (20%) and buphthalmos (10%), in comparison to 71% with leukocoria, 20% with a squint and 1% with buphthalmos among Jordanian patients (16), while in this study only 40% of patients presented with leukocoria during the war, 40% with buphthalmos and 20% with orbital disease. In fact, families in all these cases noticed the leukocoria first, and wanted to access healthcare, but because it took them 180 days to get access to care outside the siege in Gaza, the tumor growth resulted in buphthalmos. Although refugees, including Gazan patients, may encounter some challenges with financial coverage, this is generally not considered the main reason for treatment delays. Local and international funding programs typically provide sufficient support, reducing financial barriers for patients mainly children. The primary factors contributing to delayed access to care are the siege and restricted transportation, which are largely driven by the political situation.

Unfortunately, the impact of the delay in diagnosis usually reflects badly on the treatment burden. Rb management guidelines recommend that the early diagnosed tumors (group A and some group B tumors) can be managed with focal therapy even without chemotherapy, while more advanced tumors need chemotherapy and sometimes radiation and/or enucleation, so the more advanced tumor at diagnosis the more treatment burden for the patient. Enucleation is the standard treatment for group E eyes due to their high metastatic risk and is usually acceptable when the child has a functioning fellow eye (11, 24, 25). In this study, 57% of eyes diagnosed during the war required primary enucleation, compared to only 20% of eyes before the war; a hard decision that has to be accepted by the family. Although this difference was not statistically significant (p=0.144), most likely because of the small number of patients, the trend clearly shows a higher chance of eye loss due to delayed access to health care.

Furthermore, many children had already lost vision in the other eye after previous enucleation, or they presented with very advanced intraocular disease or even extraocular extension. In these situations, physicians were often forced to offer conservative treatment for group E eyes, even though the international standard is to perform primary enucleation (26), because group E eyes are known to carry a high chance of having high pathological features that increase the risk of metastasis (11, 27). The decision to avoid enucleation was not a matter of preference, but rather a desperate attempt to preserve any small amount of vision that could improve the child’s quality of life if survival was achieved. These choices also reflect the difficult reality in Gaza, where blindness or disability places an enormous extra burden on families already struggling with war, siege, and the destruction of health services. Physicians were therefore face difficult decisions, having to balance the risk of cancer spread against the hope of preserving vision in children who might otherwise face a lifetime of blindness in very harsh living conditions. This experience demonstrates how war and siege can disrupt evidence-based cancer care, forcing doctors to make decisions based not only on medical knowledge but also on political, social, and humanitarian barriers that prevent timely referral and access to proper treatment.

Our findings demonstrate a strong association between war-related delays in access to care and adverse retinoblastoma outcomes, rather than a direct causal relationship. The external validity of these findings is most relevant to settings affected by armed conflict, siege, or severe disruption of healthcare systems. The results may not be generalizable to regions with stable access to timely diagnosis and specialized pediatric oncology care. Therefore, beyond the medical and humanitarian challenges faced by local physicians, the responsibility of the international community is critical in such crises. International health and human rights organizations have emphasized that children with life-threatening but curable diseases such as Rb must be considered protected populations in times of armed conflict (28, 29). Ensuring protected humanitarian corridors, safe medical evacuation, and guaranteed access to specialized cancer care should not depend on political negotiations but on universal human rights. The United Nations Convention on the Rights of the Child obligates states to provide children with the highest attainable standard of health, regardless of political or military context (30). In practice, this requires stronger advocacy by global oncology societies, international non-governmental organizations, and diplomatic bodies to pressure decision-makers to secure timely passage of patients for life-saving treatment. In addition, collaborative networks in pediatric oncology should expand cross-border support, telemedicine, and resource sharing to mitigate the devastating impact of siege and conflict on cancer outcomes. Without such coordinated international action, treatment delays and preventable mortality among children with curable cancers like Rb will remain inevitable consequences of political inaction.

The limitations of this study include the small sample size, the relatively short follow-up period, and its retrospective design. In addition, the cohort represents only children with retinoblastoma from Gaza who were able to receive treatment in Jordan during the war; we had no access to data on any patients who may have been treated in other countries, which may introduce selection bias. Furthermore, lag time was based on parental recall as documented in the medical records, which may introduce recall bias, and other unmeasured factors related to wartime conditions—such as nutritional compromise, psychosocial stress, and overall health deterioration—may have contributed to the observed disease severity and outcomes.

In conclusion, this study highlights the profound impact of prolonged war and restricted access to healthcare on retinoblastoma outcomes, leading to delayed diagnosis, more advanced disease at presentation, and significantly reduced opportunities for both survival and vision preservation. Although retinoblastoma is a highly curable malignancy with timely treatment, disruptions in healthcare delivery can substantially worsen prognosis. These findings underscore the critical importance of ensuring uninterrupted access to specialized pediatric oncology services, even in conflict settings, to prevent avoidable morbidity and mortality. Urgent efforts are needed to maintain safe pathways for diagnosis and treatment to protect the lives and vision of affected children.

## Data Availability

The original contributions presented in the study are included in the article/supplementary material. Further inquiries can be directed to the corresponding author.
